# Supplementary material for: Enhancement of Photosynthetic Iron-Use Efficiency Is an Important Trait of Hordeum vulgare for Adaptation of Photosystems to Iron Deficiency
Source: Plants (Basel). 2021 Jan 25;10(2):234. doi: 10.3390/plants10020234 (PMC7911540; doi:10.3390/plants10020234)
Supplement: Supplementary file 1 [file plants-10-00234-s001.zip › Supplmental fileüQSaito/Supplemental Figures_Saito_Proofreading.pdf]

Figure S1

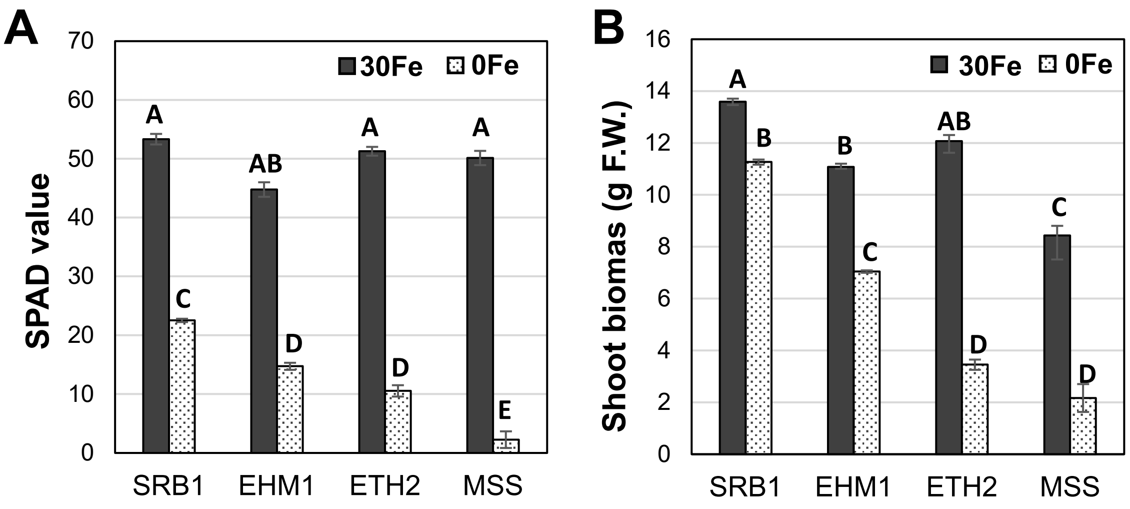

Figure S1. Fe-deficiency tolerance among four representative barley varieties. SPAD value of young developed leaves (A) and shoot fresh weight (B) of four barley varieties grown under 30 µM Fe-sufficient (30Fe) and 0 µM Fe-deficient (0Fe) condition for 16 days after 11-12 days of pre-culture with 30 µM Fe. The significant differences among all data were tested using Tukey's multiple test (N=3 ± standard error, $p < 0.05$, same lowercase letters indicate no significant difference). 'Sarab 1' (SRB1) and 'Ehime Hadaka 1' (EHM1) are the tolerant cultivars, and 'Ethiopia 2' (ETH2) and 'Musashinomugi' (MSS) are the susceptible barley.

Figure S2

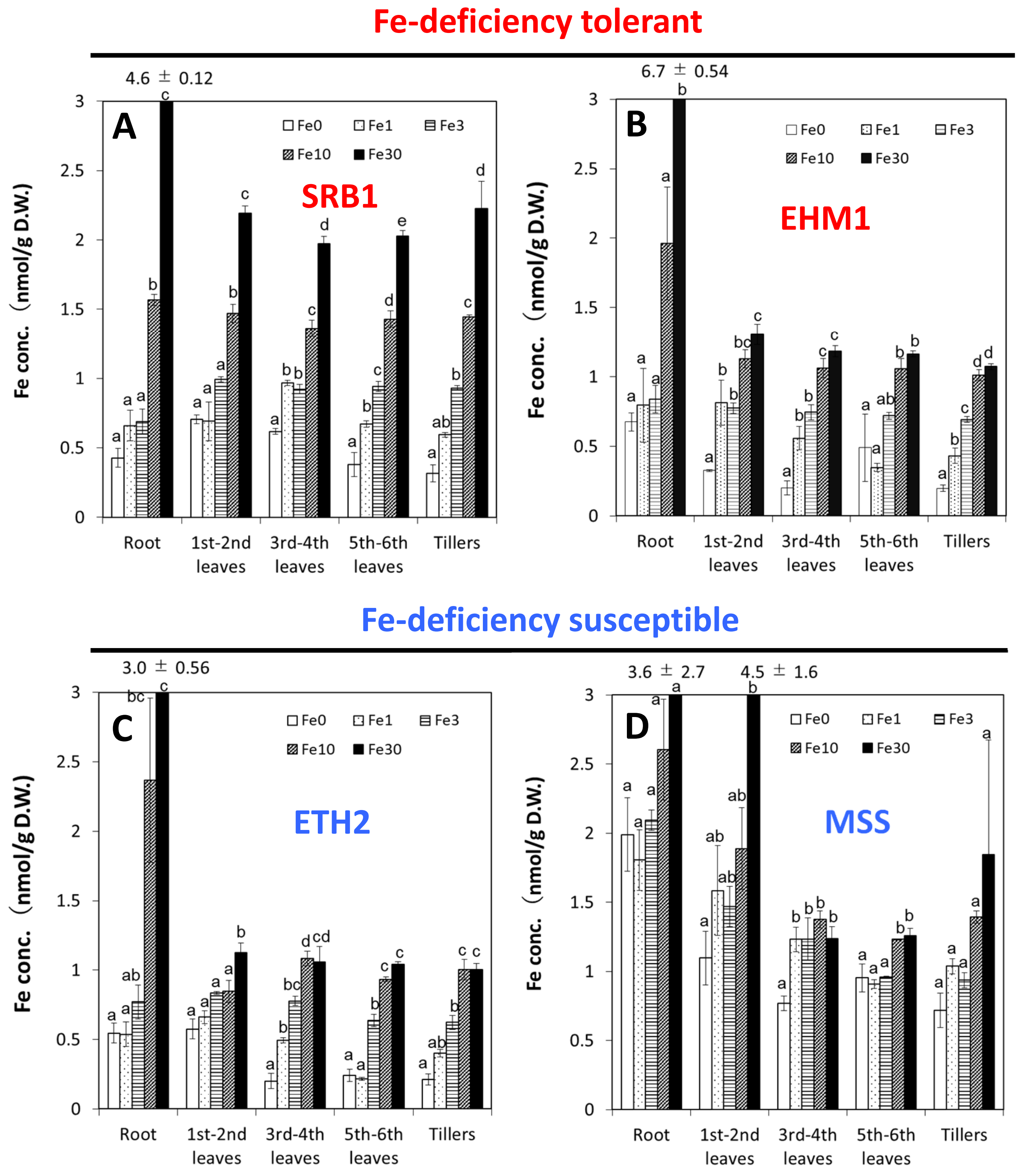

Figure S2. Comparison of the Fe concentration of four representative barley varieties showing different tolerance of Fe-deficiency. Fe concentration in each tissue of four barley varieties grown hydroponically under five Fe concentration (0, 1, 3, 10, and 30 μM) for 16 days are shown as mean of three independent experiments, with error bars indicating standard errors. Sarab 1 (SRB1) (A) and Ehime Hadaka 1 (EHM1) (B), Ethiopia 2 (ETH2) (C), and Musashinomugi (MSS) (D). The significant differences between treatments were tested using Tukey's multiple tests ($p < 0.05$, same lowercase letters indicate no significant difference).

Figure S3

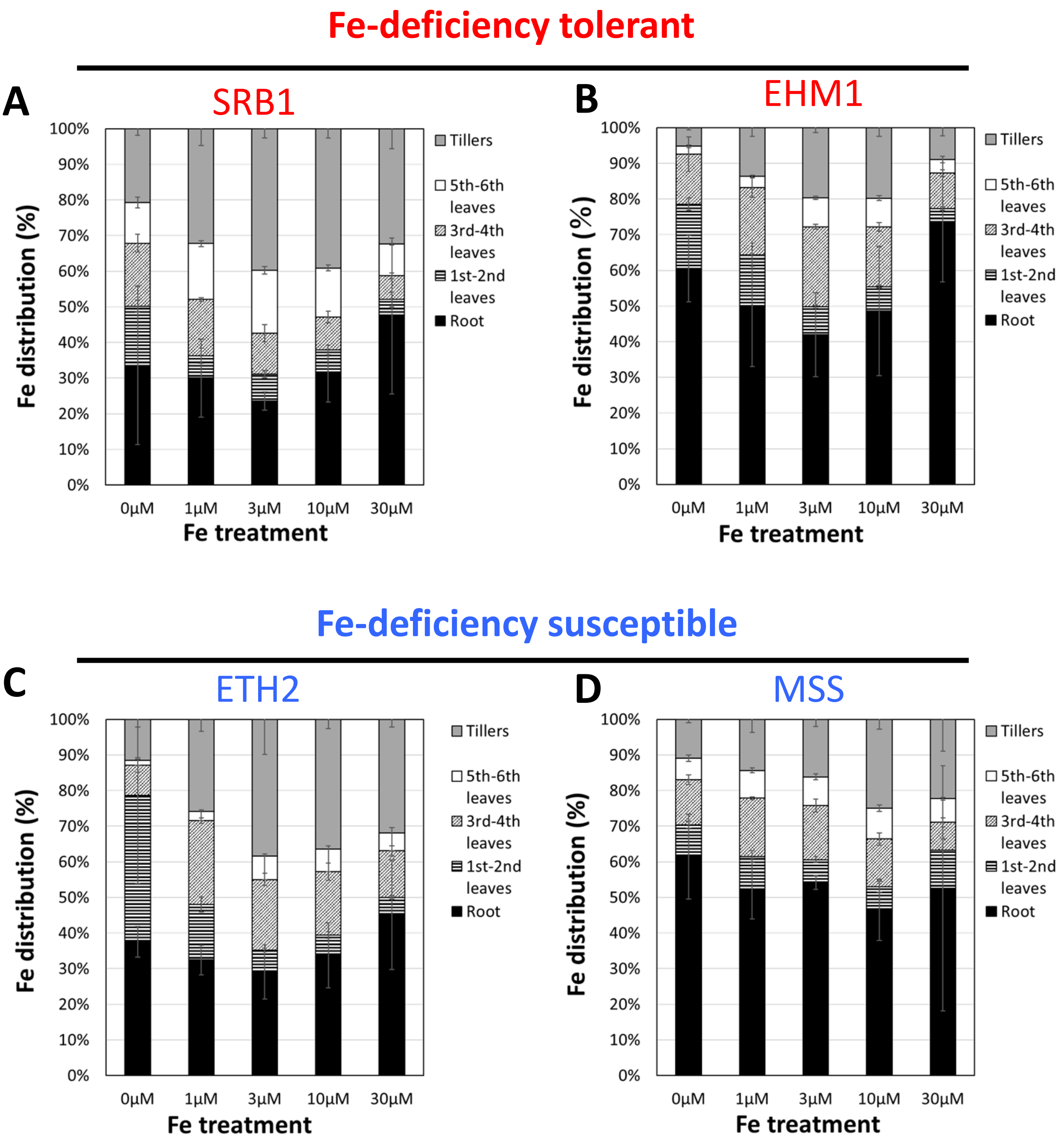

Figure S3. Comparison of the Fe distribution of four representative barley varieties showing different tolerance of Fe-deficiency. Fe distribution pattern in each tissue of four barley varieties grown hydroponically under five Fe concentrations (0, 1, 3, 10, and 30 μM) for 16 days are shown as mean of three independent experiments, with error bars indicating standard errors. 'Sarab 1' (SRB1) (A) and 'Ehime Hadaka 1' (EHM1) (B), 'Ethiopia 2' (ETH2) (C), and 'Musashinomugi' (MSS) (D). The Fe content of each tissue was calculated by multiplying the Fe concentration by the dry weight. The percentage of Fe in each tissue is shown as 100% of the total Fe in the plant.

Figure S4

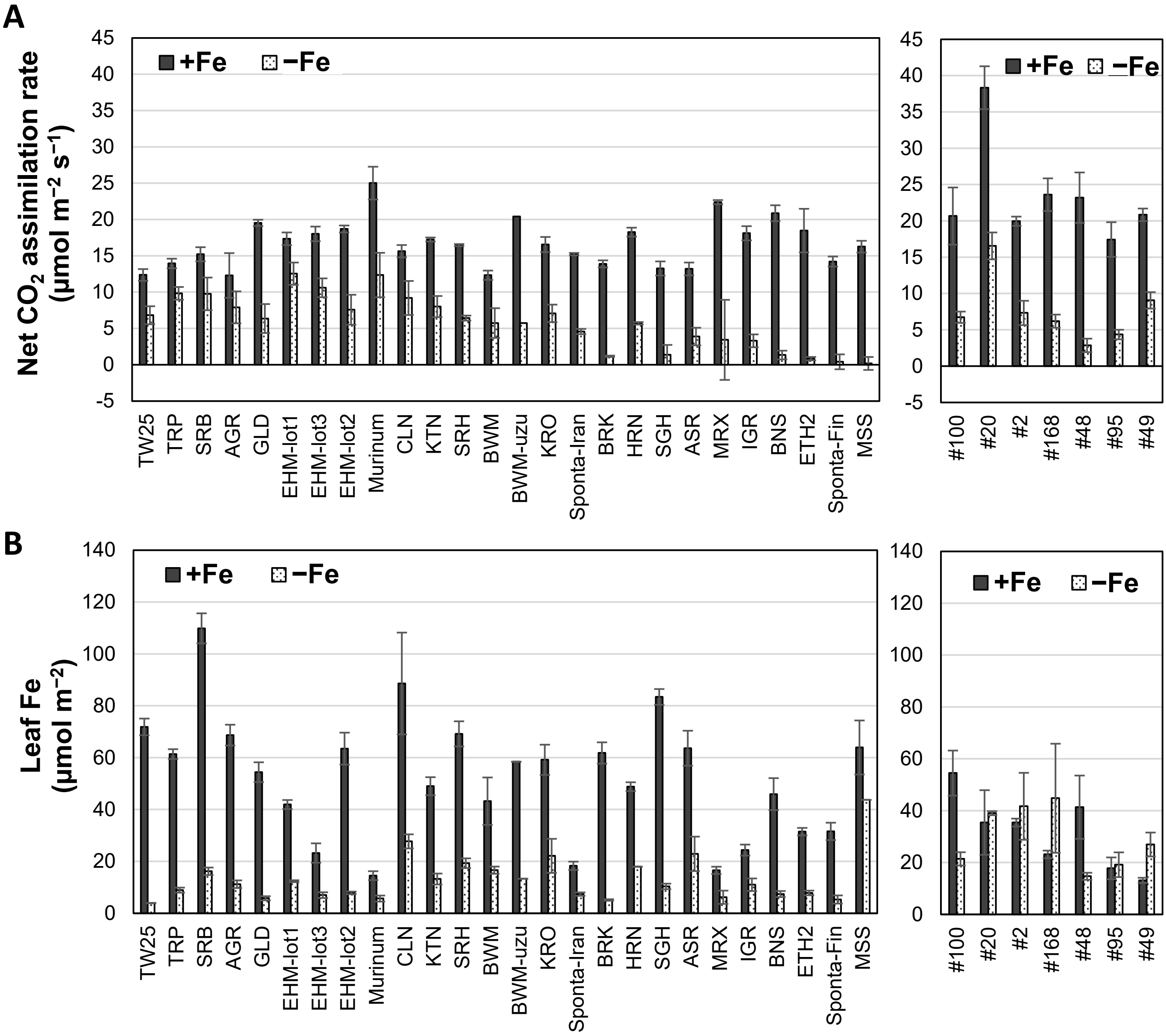

Figure S4. Raw data of CO_2 assimilation rate and leaf Fe concentration in the leaves of barley and sorghum. (A) Net CO_2 assimilation rate. (B) Fe concentrations in leaf. Data obtained plants grown under Fe-sufficient (+Fe) or Fe-deficient conditions (−Fe) to calculate PIUE as shown in Figure 4. Data are means of three replications, with error bars indicating standard errors. EHM1 as a representative variety was grown at different times to obtain data in triplicate. They are denoted as EHM1-lot1, EHM1-lot2, and EHM1-lot3. Abbreviations of plant variety names are summarized below; ‘Ehime Hadaka 1’ (EHM1), ‘Shiro Hadaka 1’ (SRH1), ‘Kairyo Ogara’ (KRO), ‘Haruna Nijo’ (HRN), ‘Akashinriki’ (ASR), ‘Saga Hadaka 1’ (SGH1), ‘Musashinomugi’ (MSS), ‘Colonial’ (CLN), ‘Bowman’ (BWM), ‘Bowman near-isogenic line *uzu1.a’* (BWM-uzu), ‘Bonus (BNS), ‘Igri’ (IGR), ‘Barke’ (BRK), ‘Morex’ (MRX), ‘Golden Promise’ (GLD), ‘Tripoli’ (TRP), ‘Sarab 1’ (SRB1), ‘Katana 1’ (KTN1), ‘Tibet White 25’ (TW25), ‘Ethiopia 2’ (ETH2), ‘Agriochriton’ (AGR), ‘Spontaneum’ originated from Iran (Sponta-Iran), ‘Spontaneum’ originated from Finland (Sponta-Fin), and *Hordeum murinum* L.(Murinum).

Figure S5

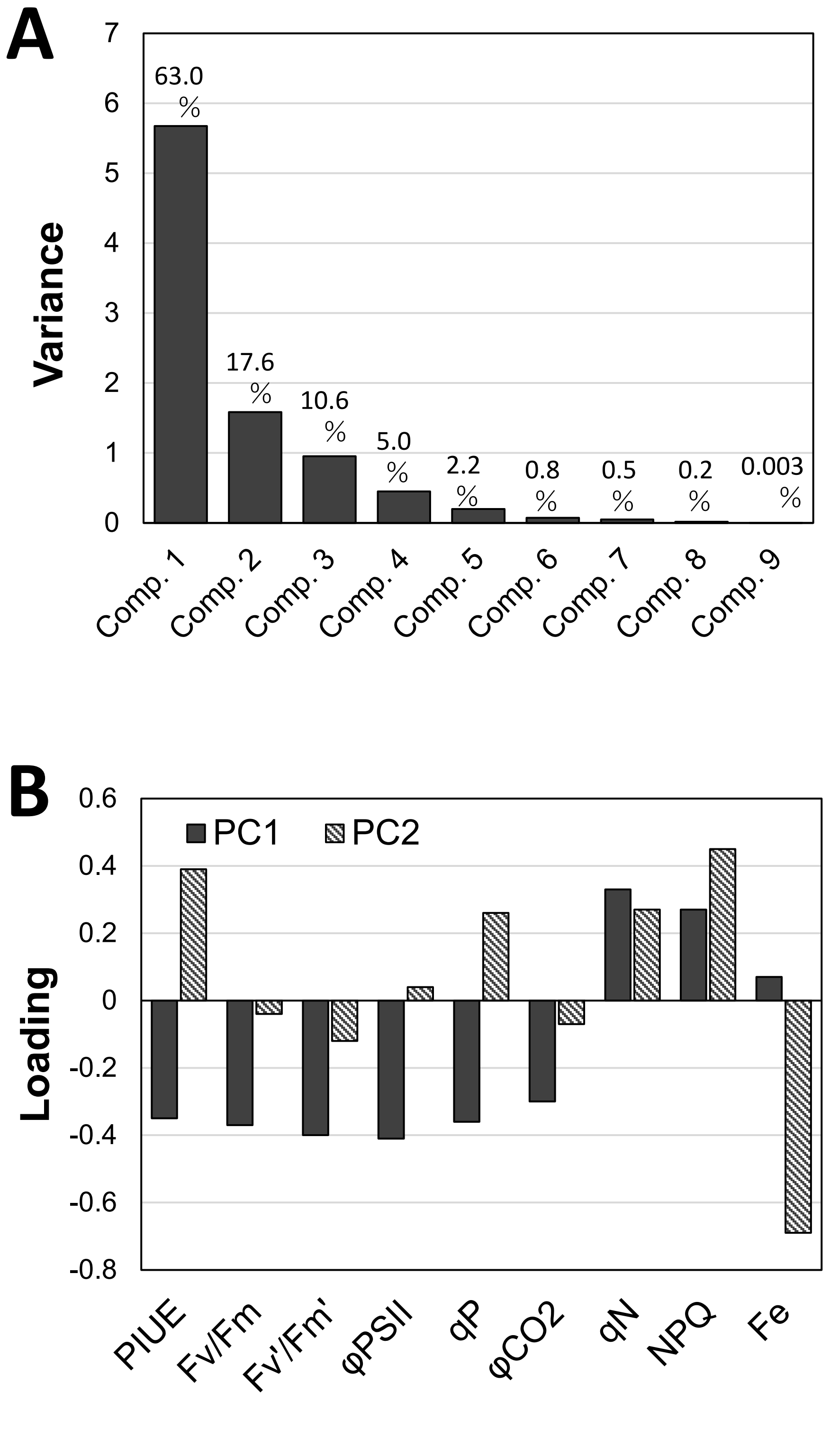

Figure S5. The proportion of variance and the loadings of individual variables in Principle component analysis (PCA) analysis. PCA was performed for nine variables in the dataset of 18 barley varieties. (A) The proportion of variance in each component (Comp.1 to Comp. 9). Percentages are indicated on the top of each column. (B) Loadings of 9 variables in PC1 and PC2 to create the loading plot in Fig. 7B.

Figure S6

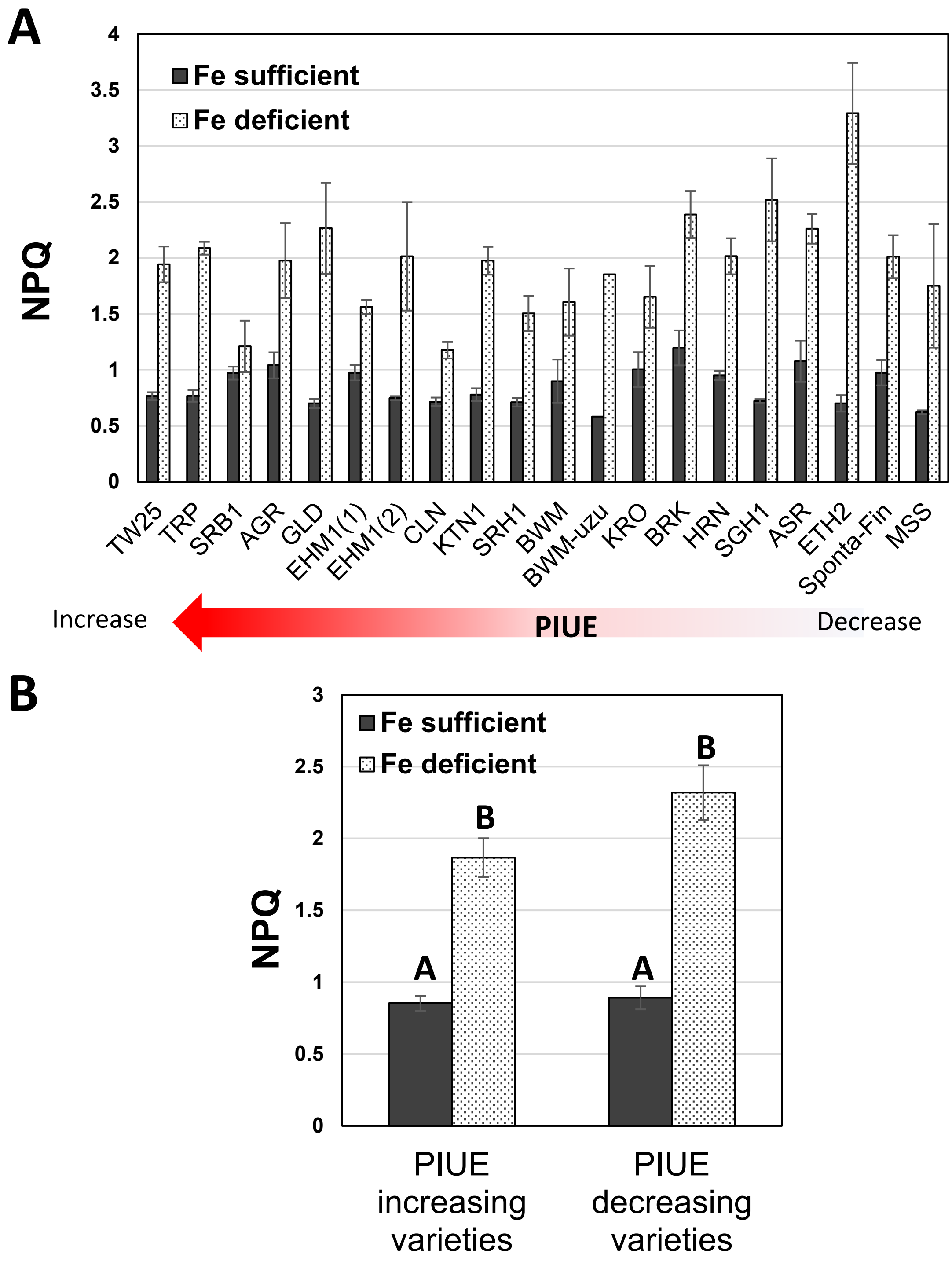

Figure S6. Nonphotochemical quenching (NPQ) among barley in young developed leaves of plants grown under Fe-sufficient or Fe-deficient conditions. Data are means of three replications, with error bars indicating standard errors. Abbreviations of plant variety names are summarized below: 'Ehime Hadaka 1' (EHM1), 'Shiro Hadaka 1' (SRH1), 'Kairyo Ogara' (KRO), 'Haruna Nijo' (HRN), 'Akashinriki' (ASR), 'Saga Hadaka 1' (SGH1), 'Musashinomugi' (MSS), 'Colonial' (CLN), 'Bowman' (BWM), 'Bowman near-isogenic line *uzu1.a*' (BWM-uzu), 'Golden Promise' (GLD), 'Tripoli' (TRP), 'Sarab 1' (SRB1), 'Katana 1' (KTN1), 'Tibet White 25' (TW25), 'Ethiopia 2' (ETH2), 'Agriochriton' (AGR), 'Spontaneum' originated from Finland (Sponta-Fin). Note that the data is a comparison within barley varieties. As shown here, NPQ induction is a universal property of barley species to protect from photosystems under Fe-deficient condition. However, it is not an explanatory variable of the PIUE changes within barley varieties.
